# Sequential Patterning of Photoresponsive Hydrogels Directs Crypt Fission and Reveals the Role of Epithelial Curvature on Fission Symmetry

**DOI:** 10.1002/advs.77397

**Published:** 2026-08-29

**Authors:** Delaney L. McNally, Kaustav Bera, Nolan R. Petrich, Alex Khang, Marilyne Coulombe, David Castillo‐Azofeifa, Ophir D. Klein, Peter J. Dempsey, Kristi S. Anseth

**Affiliations:** ^1^ Department of Chemical and Biological Engineering University of Colorado Boulder Boulder Colorado USA; ^2^ BioFrontiers Institute University of Colorado Boulder Boulder Colorado USA; ^3^ Section of Developmental Biology Department of Pediatrics University of Colorado Denver Colorado USA; ^4^ Program in Craniofacial Biology and Department of Orofacial Sciences University of California San Francisco California USA; ^5^ Department of Pediatrics Cedars‐Sinai Guerin Children's Los Angeles California USA

**Keywords:** crypt fission, hydrogels, myosin, organoids, photodegradation, photopatterning, tissue regeneration

## Abstract

Fission increases the number of crypts in the intestine during neonatal growth and also restores crypt density after injury by bifurcation of a pre‐existing parent crypt into daughter crypts. While fission is typically symmetric in healthy crypts, it is more asymmetric in diseases, and the relationship between parent crypt shape and daughter crypt (a)symmetry is difficult to study as crypt budding and fission are stochastic in organoid models and difficult to control in vivo. Here, a photoresponsive hydrogel is introduced to spatiotemporally control daughter crypt emergence from mature parent crypts in intestinal organoids, enabling longitudinal tracking of crypt bifurcation in vitro. Variation of the photopatterned dimensions tunes parent crypt shape and reveals that both fission efficiency and crypt symmetry depend on parent crypt geometry. Epithelial boundary analysis identified parent crypt curvature as a key factor influencing daughter crypt symmetry. High‐curvature or narrow crypts yielded symmetric daughter crypts, whereas wider parent crypts with lower epithelial curvature generated progressively more asymmetric crypts. Mechanistically, non‐muscle myosin IIA acts as one key regulator of crypt symmetry. Overall, this work introduces a reproducible and spatiotemporally controllable in vitro model of crypt fission, allowing identification of mechanical determinants of fission that influence intestinal regeneration and development.

## Graphical abstract

Deterministic crypt fission is achieved in intestinal organoids using photopatternable hydrogels with spatiotemporal control. Daughter‐crypt symmetry is shown to depend on parent‐crypt geometry and epithelial curvature, while myosin‐dependent mechanosensing links tissue shape to regenerative outcomes. The work establishes a controllable in vitro model for studying intestinal crypt fission.

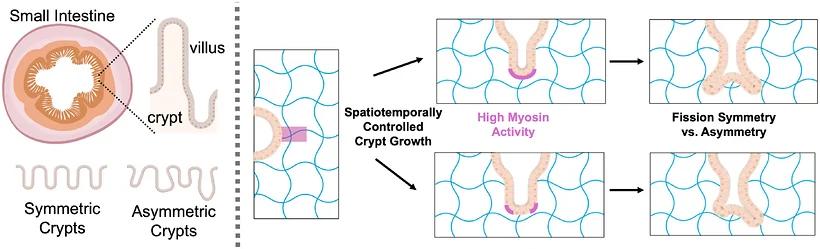

## 1 Introduction

The intestinal epithelium plays many critical functions, including the regulation of nutrient absorption and maintenance of barrier properties against mechanical, chemical, and microbial agents in the intestinal lumen. To perform these functions, the small intestine epithelium is composed of a variety of specialized cells organized in repeating crypt–villus units [1], and its integrity is maintained by the division and differentiation of crypt intestinal stem cells (ISCs) that replenish the epithelium every 5–7 days [2, 3]. While ISC division and differentiation can sustain cellular turnover during homeostasis in adult mammals, organ development and regeneration require rapid expansion in the number of crypts. This expansion is achieved primarily through a process called fission, where a pre‐existing parent crypt divides symmetrically to generate two daughter crypts of nearly equal length, which doubles the crypt number [4].

Crypt fission occurs at very low levels in healthy adults but becomes elevated when rapid expansion or restoration of crypt density is required (e.g., during neonatal growth or epithelial regeneration after intestinal injury) [4, 5]. Crypt fission is also known to become more frequent and asymmetric during pathological conditions such as inflammatory bowel disease (IBD) or colorectal cancer [6, 7, 8, 9]. Thus, deciphering how crypt fission is regulated would be beneficial to better understand the mechanisms that support healthy homeostasis, promote tissue regeneration, or prevent aberrant crypt architectures that occur in disease.

Despite the identification of signaling pathways associated with crypt morphogenesis and regeneration, key biophysical principles that govern how a parent crypt transitions into daughter crypts remain unclear. For example, does parent crypt geometry influence the efficiency of fission and the symmetry of the resulting daughter crypts, and if so, how? This question is motivated by a growing body of work supporting a form‐function relationship in the intestine [10, 11], where tissue geometry controls ISC maturation [12], fate [13, 14], and spatial patterning [15]. However, testing geometry‐driven control in the specific context of crypt fission has proven more challenging, in part because existing in vitro and in vivo models provide limited tunability over crypt shape and tracking of the same crypt throughout bifurcation.

To date, crypt fission has been inferred from fixed human and animal tissue specimens by identifying invaginations within longitudinal cross‐sections of crypts [4, 16]. More recently, intravital imaging approaches, like abdominal imaging windows implanted in mice, have allowed temporal tracking of crypts [3, 17], but these methods require complex surgeries and the epithelial behavior cannot be isolated from signaling from the surrounding stromal and mesenchymal components. In vitro, ISC‐derived intestinal organoids have provided a useful model of the intestinal epithelium [18]. Organoids recapitulate key features of the native tissue, including cell fate transitions and self‐organization into functional domains (e.g., crypt‐like buds), and have facilitated studies into crypt fission [18, 19, 20, 21, 22]. However, both crypt formation and crypt fission occur stochastically in organoid models, which can limit mechanistic studies. With this in mind, we sought to develop a method to perturb systematically the parent crypt geometry in intestinal organoids, control the timing of daughter crypt emergence, and quantitatively relate epithelial shape to fission outcomes.

Our approach exploits photopatternable poly(ethylene glycol) (PEG)‐based hydrogels that allow for the growth and differentiation of intestinal organoids with deterministic crypt morphogenesis via spatiotemporal softening of the organoid microenvironment [13, 14, 15]. To complement our prior work, we introduce a new method to study crypt fission from pre‐existing parent crypts, in which the shape and timing of daughter crypt formation can be precisely defined. This reductionist system isolates epithelial‐intrinsic contributions while enabling longitudinal tracking of the same crypt through bifurcation, thereby allowing us to directly investigate how parent crypt geometry and curvature‐induced mechanical forces regulate fission.

A two‐step, sequential photopatterning is used to first form parent crypts and subsequently direct the formation of daughter crypts from mature parent crypts. We then identify patterning conditions that reproducibly induce crypt fission with high efficiency and show that both fission efficiency and daughter‐crypt symmetry depend on the parent‐crypt dimensions and distal curvature. Leveraging Fourier‐based epithelial boundary analysis, we demonstrate that fission symmetry correlates with the parent crypt curvature independent of biochemical conditions, suggesting curvature as a predictive parameter for regenerative morphogenesis. Since curvature influences mechanical signaling in the epithelium, we then used an organoid model that allowed for inducible myosin reduction. Results implicate non‐muscle myosin IIA as a regulator of daughter crypt symmetry, supporting a role for myosin‐dependent mechanosensing in controlling epithelial‐level homeostasis during crypt bifurcation.

## 2 Results

### 2.1 Photodegradable Hydrogels Allow Sequential Photopatterning of Organoids to Initiate Crypt Bifurcation

A previously developed hydrogel system [13, 14, 15] was used to encapsulate organoids derived from murine ISCs and subsequently direct primary crypt formation, as well as initiate division into two daughter buds (i.e., fission) in a sequential patterning process. Specifically, a photoresponsive PEG hydrogel was prepared via a Strain‐Promoted Azide‐Alkyne Cycloaddition (SPAAC) reaction of two 40 kDa 8‐arm PEG macromers, one end‐functionalized with dibenzocyclooctyne (DBCO) and the other with nitrobenzyl‐ether azide (NBA) (Figure 1a). Upon mixing the macromers at room temperature, the DBCO groups and azide groups undergo a spontaneous reaction to form triazole crosslinks between the PEG macromers. PEG hydrogels with 2.5 wt.% macromers and a final shear storage modulus (G’) of ∼500 Pa were used to encapsulate organoids, along with 0.8 mM Arg‐Gly‐Asp (RGD) peptide and 0.14 mg/mL laminin mixed in the pre‐gel solution to promote organoid survival and differentiation in accordance with previous reports [13, 23]. Specific regions within the hydrogel were then softened by raster scanning with a 405 nm single‐photon confocal laser light operated at 1.79 mW power, irradiating each pixel of 420 nm size for 12.64 µs. Light exposure cleaves the nitrobenzyl‐ether linker and lowers the crosslinking density, softening the hydrogel at regions defined in the X‐Y directions by the photopatterning dimensions [24, 25] (Figure 1b). Prior work has shown that the selected light dose is effective in softening the hydrogel matrix and leads to morphological changes in organoids without affecting viability [13, 14, 15] (see Section 4 for detailed method).

**FIGURE 1:**
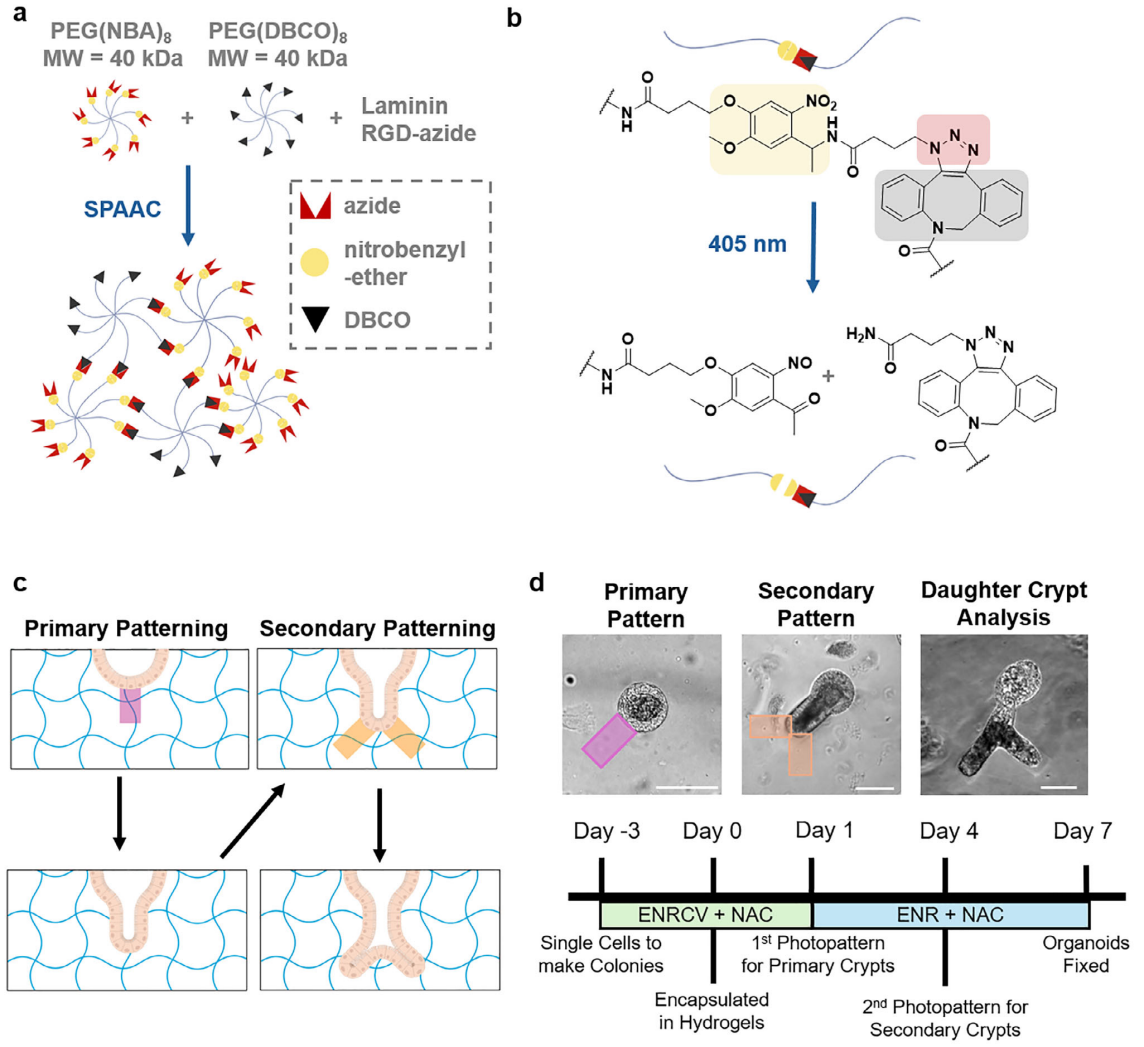
Sequential patterning of organoids in photodegradable hydrogels to spatiotemporally control in vitro crypt fission: (a) Schematic of 40 kDa 8‐arm PEG macromers functionalized with dibenzocyclooctyne (DBCO) or nitrobenzyl‐ether azide (NBA). DBCO groups and azide groups undergo a Strained‐Promoted Azide‐Alkyne Cycloaddition (SPAAC) reaction spontaneously to form triazole crosslinks between the PEG macromers. Laminin and azide‐functionalized RGD peptide were also incorporated into the hydrogel. (b) After network formation, exposure to a 405 nm laser initiated network degradation via cleavage of the nitrobenzyl‐ether group. The yellow highlighted section represents the nitrobenzyl‐ether photocleavable unit, whereas the red and grey highlighted sections represent the portions of the triazole product originating from the azide and DBCO precursors, respectively. (c) Schematic of sequential photopatterning process starting from spherical organoid colonies encapsulated in PEG hydrogel through primary and secondary patterning and subsequent formation of bifurcated crypts. Purple and orange regions indicate areas of primary and secondary photopatterning, respectively. (d) Representative images of organoids during the photopatterning process. The primary pattern (purple) was used to degrade a region close to the spherical colony to obtain the parent crypt. Then, secondary patterns (orange) were used to degrade regions at the parent crypt base to obtain daughter crypts. The schematic outlines the experimental timeline and media types. The primary patterning was performed on day one, and the secondary patterning was performed on day 4 to initiate parent crypt fission into daughter crypts. Organoids were grown until day 7 for analysis. Scale = 100 µm.

Three‐day‐old ISC colonies grown in Matrigel were encapsulated on day zero in PEG hydrogels, and the colonies were maintained in their cystic, circular morphology with stem‐promoting medium (ENRCV+Nac) for one day. Next, softened channels of specific dimensions (defined in X‐Y by the primary patterns) were created around the encapsulated organoids by exposure to 405 nm confocal laser light (Figure 1c,d), and the medium was simultaneously switched to differentiation conditions (ENR+Nac). The softened regions in the hydrogel matrix permitted outward deformation of the epithelia and subsequent symmetry breaking of spherical organoids. The organoids were then cultured for another three days, which promoted buds to grow and differentiate into fully developed intestinal crypts as previously observed [13, 14, 15]. By day four (three days after primary patterning), the crypt cells proliferated till the end of the primary pattern (Figure 1c,d and Figure S1). The timeline for parent crypt growth and media compositions is consistent with conditions used previously that allowed for robust crypt growth and differentiation in photopatternable PEG hydrogels [13, 14, 15]. Subsequently, to mimic division of parent crypts during crypt fission, we performed a second photopatterning, creating two orthogonal softened channels at the crypt base using a “V” shaped secondary pattern. Of note, the organoids were maintained in differentiation medium for another three days, until day seven, before subsequent analyses (Figure 1c,d). Thus, using sequential photopatterning, we obtained “Y” shaped final patterns around the initially spherical organoids, with parent crypts occupying the stems of the Y‐patterns and bifurcated daughter crypts growing into the two arms. The secondary patterning allowed the parent crypt to divide into two daughter crypts, which grew into the softened regions, providing a system to not only control the timing of crypt division in vitro but also the shape of the parent and daughter crypts.

### 2.2 Programmable Control of Daughter Crypt Growth by Photopattern Length

After establishing the appropriate photopatterning sequence, we next sought to identify primary and secondary pattern dimensions that would produce viable daughter crypts with high spatiotemporal control. To quantify the effect of different patterning parameters on the resulting daughter crypts, we defined “fidelity” to indicate the extent to which daughter crypt growth matched the prescribed secondary pattern length. This fidelity was defined by the ratio of the daughter crypt length (L_E_) compared to the secondary patterning length (L_P2_) (Figure 2a). First, two different pattern lengths for templating parent crypts were tested by photopatterning with primary patterns (L_P1_) of 50 and 100 µm lengths (Figure 2b), and the effect on daughter crypt fidelity was examined. Of note, the width of the primary pattern was kept constant at 50 µm, a value close to murine crypt diameters [15, 26], and the secondary pattern was fixed at 100 µm long and 40 µm wide. While both 50 and 100 µm primary pattern lengths resulted in healthy and viable daughter crypts, the daughter crypts originating from 50 µm long primary patterns experienced noticeable growth outside the specified patterned area (> 100% mean fidelity), compared to the 100 µm long patterns (Figure 2b,c). Based on these findings, the sequential patterning in the subsequent experiments was performed with a primary pattern length of 100 µm.

**FIGURE 2:**
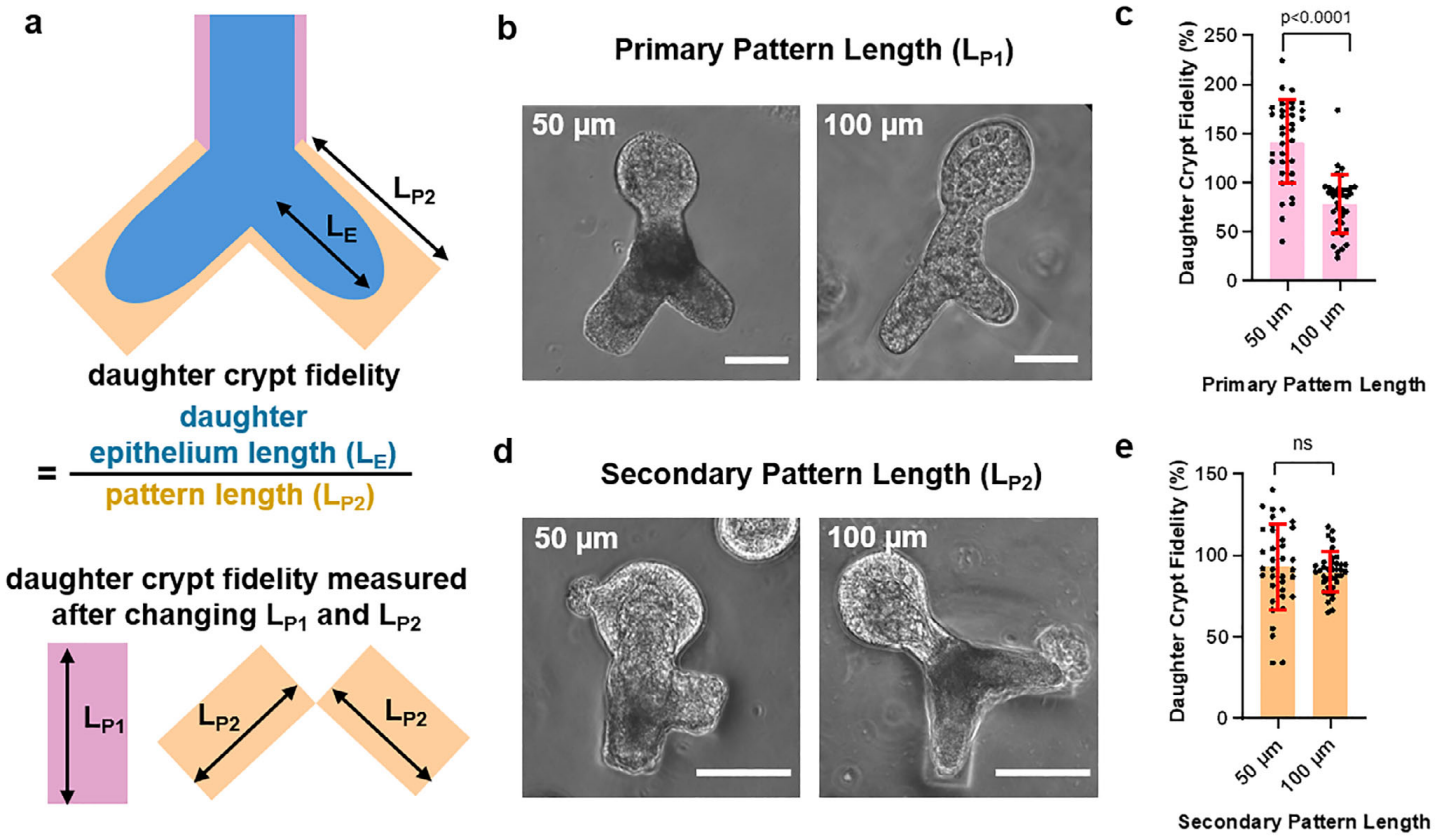
Photopattern length determines control over daughter crypt growth. (a) Schematic of daughter crypt fidelity calculation by obtaining the ratio of daughter crypt epithelial length (L_E_) over secondary pattern length (L_P2_). Daughter crypt fidelity was evaluated after changing primary (L_P1_) and secondary (L_P2_) pattern lengths. (b) Representative images of organoids with daughter crypts originating from parent crypts grown within 50 or 100 µm long primary patterns. Both conditions had 100 µm long secondary patterns. Scale = 100 µm. (c) Daughter crypt fidelity originating from 50 or 100 µm long primary patterns. For 50 and 100 µm, 34 and 36 daughter crypts were analyzed, respectively. (d) Representative images of organoids with daughter crypts grown within 50 or 100 µm long secondary patterns. Both conditions had 100 µm long primary patterns. Scale = 100 µm. (e) Daughter crypt fidelity within 50 or 100 µm long secondary patterns. For 50 and 100 µm, 36 and 37 daughter crypts were analyzed, respectively. Statistical analyses were performed using unpaired t‐tests (in c, e). Bars denote averages ± SD and points denote measurements from individual crypts.

Next, we varied the length of the secondary patterns (L_P2_) used to initiate bifurcation of the parent crypts and studied their effect on daughter crypt fidelity. The secondary pattern lengths considered were 50 and 100 µm (Figure 2d), and the width was kept constant at 40 µm. While there was no statistically significant difference in the average daughter crypt fidelity, longer patterns had less variability (σ = 26.2 vs. 12.3 for the 50 and 100 µm patterns, respectively) and thus more control over the daughter crypt dimensions (Figure 2e). Based on these results, we selected iterative patterning conditions of 100 µm long patterns for both the primary and secondary photopatterning, which were kept constant for the remainder of the experiments unless specified.

After identifying the patterning dimensions that led to consistent daughter crypts of defined dimensions at spatially directed locations, we next sought to study the effect of increasing the number of parent crypts in each organoid, and thus the number of subsequent daughter crypts, to improve experimental throughput (Figure S2a). For these experiments, we used organoids (subsequently called Defa4/tdTomato organoids) derived from Paneth cell reporter mice (*Defa4^Cre^/Rosa26^tdTomato^*) [15, 27], where Paneth cells express the fluorescent reporter tdTomato and are used to define the formation of crypts. The Defa4/tdTomato organoids were used to investigate if the number of primary patterns had any effect on parent crypt survival and differentiation (Figure S2b,c). Since we observed no difference in Paneth cell numbers between organoids with one or two parent crypts, we performed subsequent experiments by exposing each encapsulated organoid to two primary patterns of equal dimensions (Figure S2a). This allowed us to obtain two parent crypts from each organoid and thus increase the number of parent and daughter crypt data points from each organoid.

### 2.3 Photopatterned Daughter Crypts Contain Crypt‐Specific Cell Types and Divide Symmetrically

Using the photopatterning protocol that best recapitulated a reproducible crypt fission morphology, we next evaluated both cellularity and morphometrics after crypt division into daughter crypts. We observed that more than 70% of the daughter crypts contained at least one Paneth cell, indicative of functional daughter crypts (Figure 3a,b) after bifurcation. We also observed that the Paneth cells were localized to the crypt base, typically intercalated among Olfactomedin 4 (Olfm4) expressing intestinal stem cells (Figure 3c). This spatial organization is characteristic of mature intestinal crypts and indicates successful daughter crypt formation post‐secondary photopatterning of parent crypts.

**FIGURE 3:**
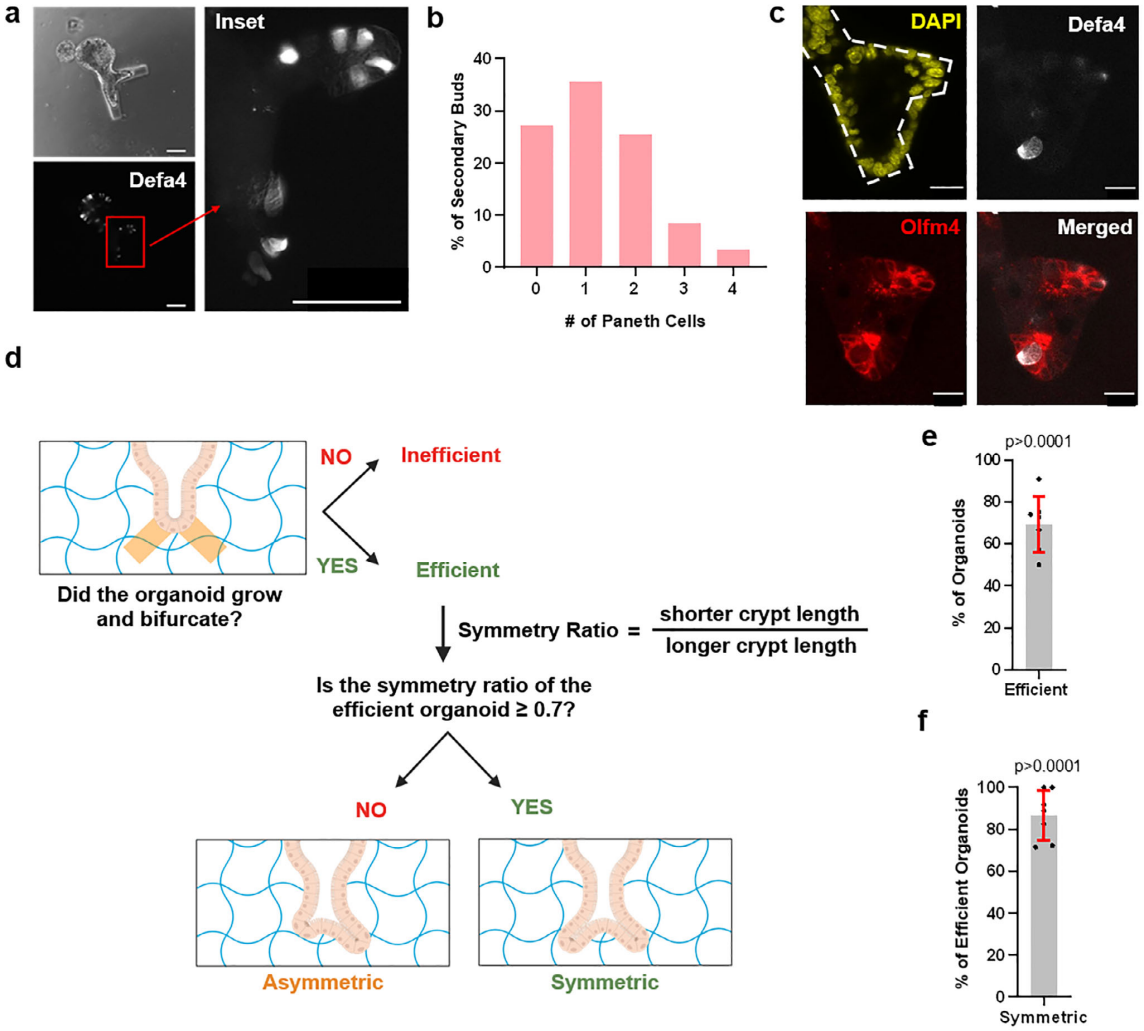
Presence of crypt‐specific cells in patterned daughter crypts. (a) Representative phase and fluorescent images of Defa4 expressing Paneth cells in bifurcated daughter crypts three days post‐fission. Scale = 100 µm. (b) Percentage of buds growing within secondary patterns with the indicated number of Paneth cells. Data from 59 organoids from 4 independent experiments. (c) Representative confocal images showing localization of stem cells (Olfm4+) and Paneth cells in daughter crypts three days post‐fission. White dotted line indicates a schematic of the bifurcated daughter crypts and the parent crypt. Paneth cells localize at the base of the crypts and are surrounded by stem cells. Scale = 20 µm. (d) Schematic of daughter crypt morphology analysis. Organoids where the parent crypt divides and grows buds into the secondary patterns were classified as efficient. All efficient organoids were then analyzed for crypt symmetry ratios to determine symmetric and asymmetric crypts. (e) Percentage of organoids efficient or inefficient in growing daughter crypts after secondary patterning. Each datapoint represents one experiment. Data from 7 experiments with 184 organoids in total. (f) Percentage of efficient organoids demonstrating symmetric or asymmetric daughter crypts. Daughter crypts with symmetry ratio ≥ 0.7 (or 70%) were considered symmetric, and the remaining were considered asymmetric. Each datapoint represents one experiment. Data are from 7 experiments with 127 organoids in total. Statistical analyses were performed using one‐sample t‐tests in (e, f), with a hypothetical mean of zero.

After confirming cellularity of the daughter crypts, we sought to further characterize the fission process based on tissue‐level crypt morphometrics. We defined crypt fission “efficiency” as the percentage of organoids that formed daughter crypts after undergoing secondary patterning (Figure 3d). Among the organoids that were efficient (i.e., formed daughter crypts), we then evaluated the daughter crypt “symmetry” as the ratio of the lengths of the shorter to the longer crypt [28] (Figure S3). A ratio of approximately 0.7 (or 70%) was considered symmetric crypt fission and typically an indicator of healthy physiology [28], whereas lower values were associated with aberrant fission, which is usually found in diseased states like IBD [29, 30]. Results revealed that a high percentage of organoids (∼70%) led to efficient daughter crypt formation in our hydrogels (Figure 3e), and of these crypts, ∼85% displayed symmetric fission (Figure 3f). Collectively, these data indicate that sequential photopatterning of intestinal organoids in PEG hydrogels produces daughter crypts with a physiological crypt cellularity with high efficiency and morphometric symmetry that resembles healthy crypt fission.

### 2.4 Parent Crypt Shape Regulates Daughter Crypt Symmetry

After establishing the efficiency of the hydrogel system to model fission within intestinal organoids, we next tested whether parent crypt geometry can regulate daughter crypt fission symmetry by varying the width of the primary pattern and thus altering the parent crypt shape. While in our experiments so far we used a primary pattern width of 50 µm to mimic the average murine crypt width [15, 26], we next generated parent crypts within primary patterns of either 50 or 70 µm width using our sequential photopatterning protocol (Figure 4a). The two different pattern widths constrained crypt growth laterally between typical to supraphysiological dimensions, while creating variation in the shape of the parent crypt (e.g., curvature of the distal region) [15]. The lengths of both primary (L_P1_) and secondary (L_P2_) patterns were maintained at 100 µm as per results obtained in Section 2.2. We quantified parent crypt shape after photopatterning by measuring epithelial curvature (κ) using a Fourier series representation of organoid geometry (Figure 4b). The narrow, 50 µm wide primary patterns produced crypts with higher average epithelial curvature at the distal end of the crypt (κ ranging from 0.04 to 0.96 µm^−1^), compared to the 70 µm wide ones (κ ranging from 0.03 to 0.34 µm^−1^) (Figure 4c). Next, we performed secondary photopatterning on these parent crypts and observed that the wider patterns (70 µm) led to a reduction in fission efficiency (Figure 4d,e) and formed daughter crypts with reduced fission symmetry (Figure 4f). However, interestingly, there was no effect of pattern width on the average daughter crypt length (Figure 4g).

**FIGURE 4:**
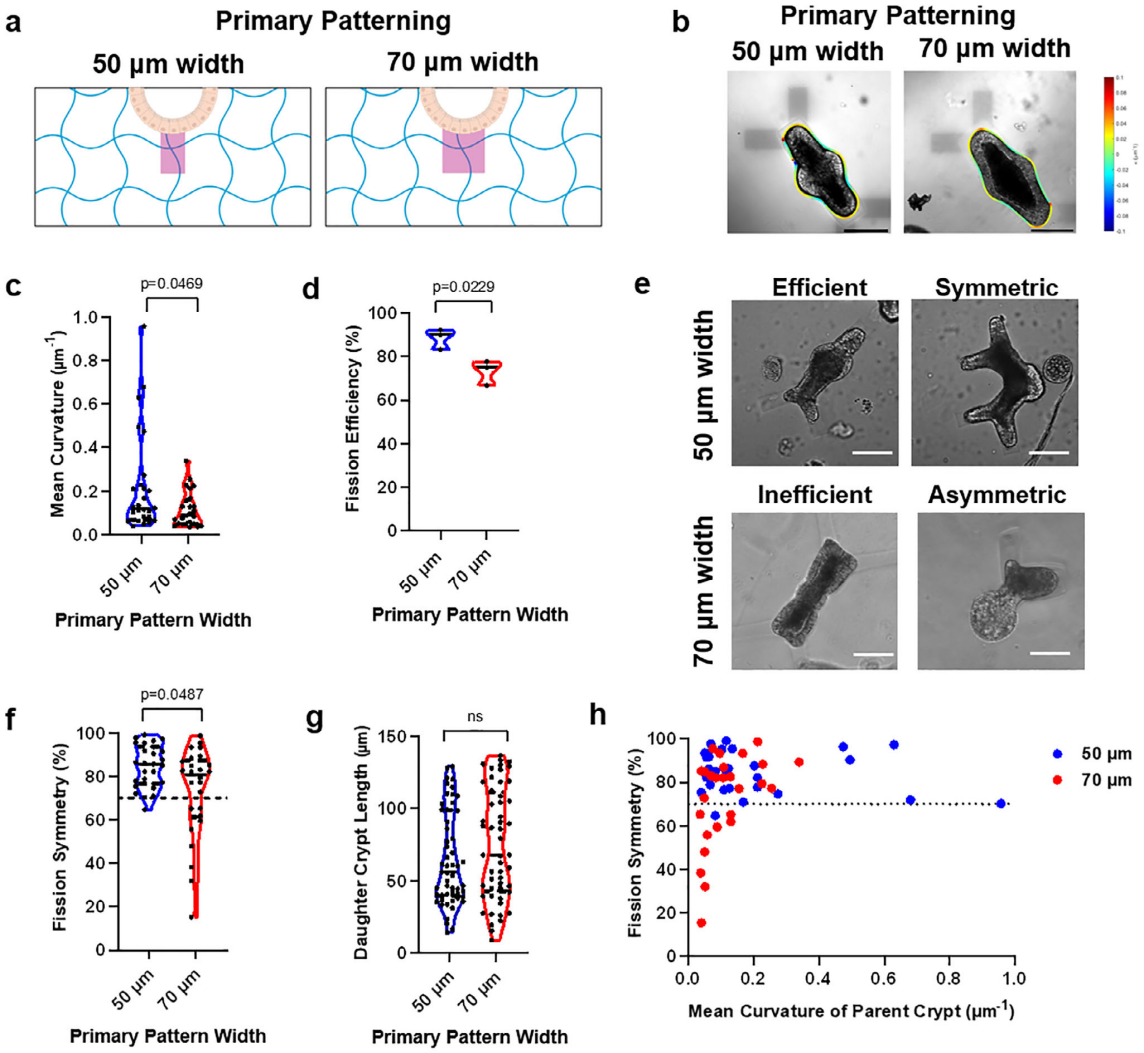
Control of parent crypt dimension using photopatterning and its effect on daughter crypt symmetry. (a) Schematic of 50 and 70 µm wide primary patterns for controlling parent crypt shape. (b) Representative images of curvature profiles overlaid on the respective organoids with parent crypts three days after 50 or 70 µm wide primary patterning. Scale = 100 µm. (c) Distribution of mean values for the top 5% of positive epithelial curvatures from organoids exposed to the indicated primary pattern width. (d) Fission efficiency percentage of organoids exposed to the indicated primary pattern width. Data from 3 experiments, and each data point represents the mean efficiency from one experiment. (e) Representative images of efficiency and symmetry trends seen in panel d for both 50 and 70 µm wide primary patterning. Scale = 100 µm. (f) Fission symmetry ratios as percentages at indicated primary pattern widths. Dotted black line denotes 70% symmetry ratio. (g) Daughter crypt lengths at indicated primary pattern widths. (h) Plot of individual curvature values for parent crypts and the corresponding post‐fission symmetry values as percentages. Blue dots correspond to 50 µm and red dots to 70 µm primary pattern widths. The dotted black line represents the healthy symmetry ratio at 70%. For (c, e–g), 26 and 25 parent crypts in 50 and 70 µm widths, respectively, were analyzed from 3 independent experiments. The black lines indicate the median (thick) and quartiles (dotted). Statistical analyses were performed using unpaired t‐tests (d and after log‐transformation in c) and Mann–Whitney tests (f, g).

We further investigated the cellular composition of daughter crypts after both symmetric and asymmetric fission by characterizing the number density of Ki67‐positive proliferative cells and differentiated Paneth cells. Interestingly, we observed no difference in Paneth cell number density between symmetric and asymmetric daughter crypts (Figure S4a), neither between the longer and shorter crypts in each case (Figure S4b,c). We saw similar results with Ki67 staining, where no changes were observed in the two daughter crypts resulting from either symmetric or asymmetric fission (Figure S5). These data suggest that once the daughter crypts are formed, they reach the same degree of compositional maturity irrespective of crypt symmetry.

Finally, to decouple the shape of the observed epithelium from the specified photopatterning dimensions within the hydrogel matrix, we longitudinally tracked daughter crypt symmetry as a function of the measured parent crypt curvature across the two primary patterning widths of 50 and 70 µm (Figure 4h). The analysis revealed a strong curvature‐associated increase in fission symmetry, with symmetry rising sharply at low curvature values, but then plateaued above the healthy threshold of 70% at higher curvature values (Figure 4h). Together, these results demonstrate that photopatterning enables deterministic control over parent‐crypt geometry, while measured curvature provides a quantitative descriptor of the actual epithelial shape achieved under each condition. Thus, parent‐crypt curvature emerges as an important geometric parameter associated with daughter‐crypt symmetry, whereas primary pattern width acts as an experimental control that biases the epithelial geometry of the parent crypt. Specifically, narrower parent crypts, which tend to exhibit higher distal curvature at the stem cell niche, are more likely to produce daughter crypts with a physiologically healthy symmetry during fission.

### 2.5 Myosin is a Regulator of Parent Crypt Curvature‐Dependent Fission Symmetry

Based on the correlation between curvature and fission efficiency and symmetry, we next sought to identify what mechanosensitive pathways might underlie the parent crypt geometry‐dependent control of fission symmetry. Additionally, the similarity in cellular composition between shorter and longer daughter crypts resulting from both symmetric and asymmetric fission (Figures S4 and S5) suggests that fission symmetry could be influenced by the epithelial state prior to daughter crypt division, i.e., at the parent crypt stage. Recent literature suggests that non‐muscle myosin II plays an important role in sensing extracellular tension and in controlling cellular contractility within single cells [31] and in intestinal epithelium [26, 32]. This motivated us to test if myosin II is also differentially activated in response to epithelial curvature changes during fission, by immunostaining for phosphorylation (Ser19) of myosin light chain 2 (pMLC) [31]. Indeed, in parent crypts generated within 50 and 70 µm wide primary patterns, we observed increased pMLC, indicative of myosin II activity, in regions of higher levels of curvature in the epithelium (Figure 5a). When the intensity of pMLC was plotted with respect to the local curvature of the epithelium, we observed a correlation with increases in epithelial curvature (Figure 5b and Figure S6a,b). These immunostaining experiments suggest that local epithelial curvature may lead to changes in myosin activity in parent crypts, which can then subsequently regulate fission symmetry.

**FIGURE 5:**
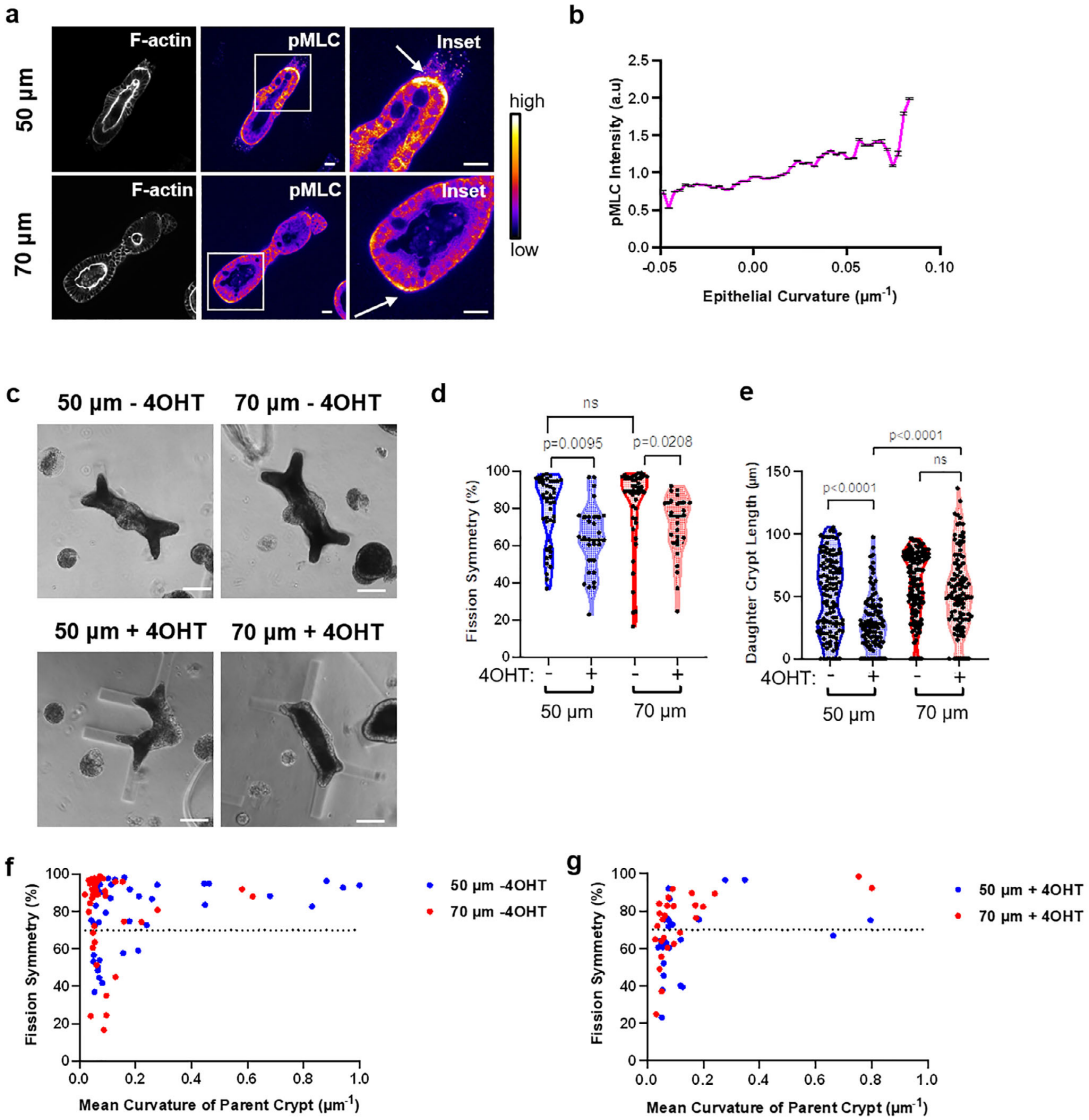
Myosin as a sensor of epithelial curvature. (a) Representative confocal images of organoids with parent crypts obtained at indicated primary pattern widths, stained for F‐actin and pMLC. Heatmap shows levels of pMLC, and white arrows indicate regions of high curvature with elevated pMLC levels. Scale = 20 µm. (b) Normalized pMLC intensity along the epithelial boundary plotted against local epithelial curvature of organoids exposed to 50 and 70 µm primary patterns. Data are the moving average ± s.e.m. for ≥ 40 organoids from 3 experiments. (c) Representative images of iMyh9 organoids with 50 and 70 µm wide primary patterns on day seven. Top panels show organoids without treatment, while bottom panels show organoids treated with 0.1 µM 4OHT starting on day three. Scale = 100 µm. (d) Fission symmetry ratios as percentages of iMyh9 organoids at indicated primary pattern widths, with or without 4OHT induction on day three after primary crypt growth. Data from 5 experiments with ≥ 86 daughter crypts per condition. (e) Daughter crypt lengths of iMyh9 organoids within indicated primary patterns, with or without 4OHT induction on day three after primary crypt growth. Data from 5 experiments with ≥ 86 daughter crypts per condition. The black lines indicate the median (thick) and quartiles (dotted). (f) Plot of individual curvature values for parent crypts in iMyh9 organoids and the corresponding post‐fission symmetry values as a percentage. Blue dots correspond to 50 µm and red dots to 70 µm primary pattern widths. In total, 57 parent crypts were analyzed from 3 independent experiments. (f, g) Plot of individual curvature values for parent crypts at both 50 and 70 µm pattern widths, and the corresponding post‐fission symmetry values as a percentage for iMyh9 organoids without (f) and with 4OHT treatment (g). In (g), the iMyh9 organoids were treated with 0.1 µM 4OHT on day three. Statistical analyses were performed using Kruskal–Wallis tests followed by Dunn's tests (d, e).

Since non‐muscle myosin heavy chain IIA (Myh9) is one of the two epithelial isoforms of non‐muscle myosin II, we asked if Myh9‐mediated contractility might be a plausible driver of force generation within the intestinal epithelium [26, 33]. We began by testing the role of crypt shape on fission symmetry in intestinal organoids isolated from iMyh9 (Villin‐CreER;Myh9^flox/flox^;Rosa26^mTmG^) mice (Figure 5c), where treatment with 4‐hydroxytamoxifen (4OHT) results in a conditional knockout of Myh9. Organoids were grown, and parent crypts were allowed to develop normally for two days without 4OHT treatment (Figure S7). Reduction of Myh9 levels during crypt fission through 4OHT (0.1 µM) treatment produced daughter crypts with significantly lower symmetry ratios from parent crypts generated using both 50 and 70 µm wide primary patterns (Figure 5d). In addition, 4OHT treatment led to shorter daughter crypts, but only in the 50 µm wide parent‐crypt condition (Figure 5e). Notably, the overall relationship between daughter‐crypt symmetry and parent‐crypt curvature was maintained in iMyh9 organoids regardless of 4OHT treatment (Figure 5f,g). Together, these data suggest that Myh9‐dependent contractility contributes to fission progression and may be especially important in narrower crypts, which typically exhibit higher epithelial curvature.

## 3 Discussion

Crypt fission is an important developmental and regenerative process necessary for neonatal intestinal growth, for maintaining homeostasis in adults, and for restoring epithelial cell homeostasis after injury [4, 5]. Although crypt fission becomes irregular and less symmetric during diseases such as IBD and colon cancer [6, 7, 8, 9], less is known about how the parent crypt shape might influence the morphology of daughter crypts. This question seems compelling given observations of altered intestinal tissue morphometrics from patients with IBD [34, 35] and colon cancer [36]. Considering the emerging form–function relationship in the intestine, it is tempting to hypothesize that changes in crypt morphology could directly bias daughter crypt formation, irrespective of the extrinsic non‐epithelial cues. Thus, in this study we were specifically interested in identifying how alterations in epithelial shape might change the dynamics of daughter crypt formation within an epithelial‐only system, without confounding contributions from the mesenchyme.

To address this question, we developed an in vitro photopatternable hydrogel system that enables microscopic control over parent and daughter crypt shapes, and the timing of parent crypt bifurcation. By varying the dimensions of the regions in the hydrogel exposed to light, we altered parent epithelial geometry in a controlled manner and then tracked the morphometrics of daughter crypts arising from each parent crypt. By mapping fission symmetry to parent crypt curvature, we uncovered a previously unrecognized relationship between parent crypt shape and daughter crypt fission symmetry under otherwise uniform biochemical cues (Figure 6). Specifically, parent crypts above a curvature threshold or below a width threshold produced symmetric daughter crypts. In contrast, wider parent crypts, which correspond to lower epithelial curvature, produced increasingly asymmetric daughter crypts, with a monotonic decrease in symmetry as curvature decreased. Thus, our finding that fission symmetry is linked to parent crypt curvature, independent of biochemical conditions, establishes epithelial curvature as a predictive parameter for regenerative morphogenesis.

**FIGURE 6:**
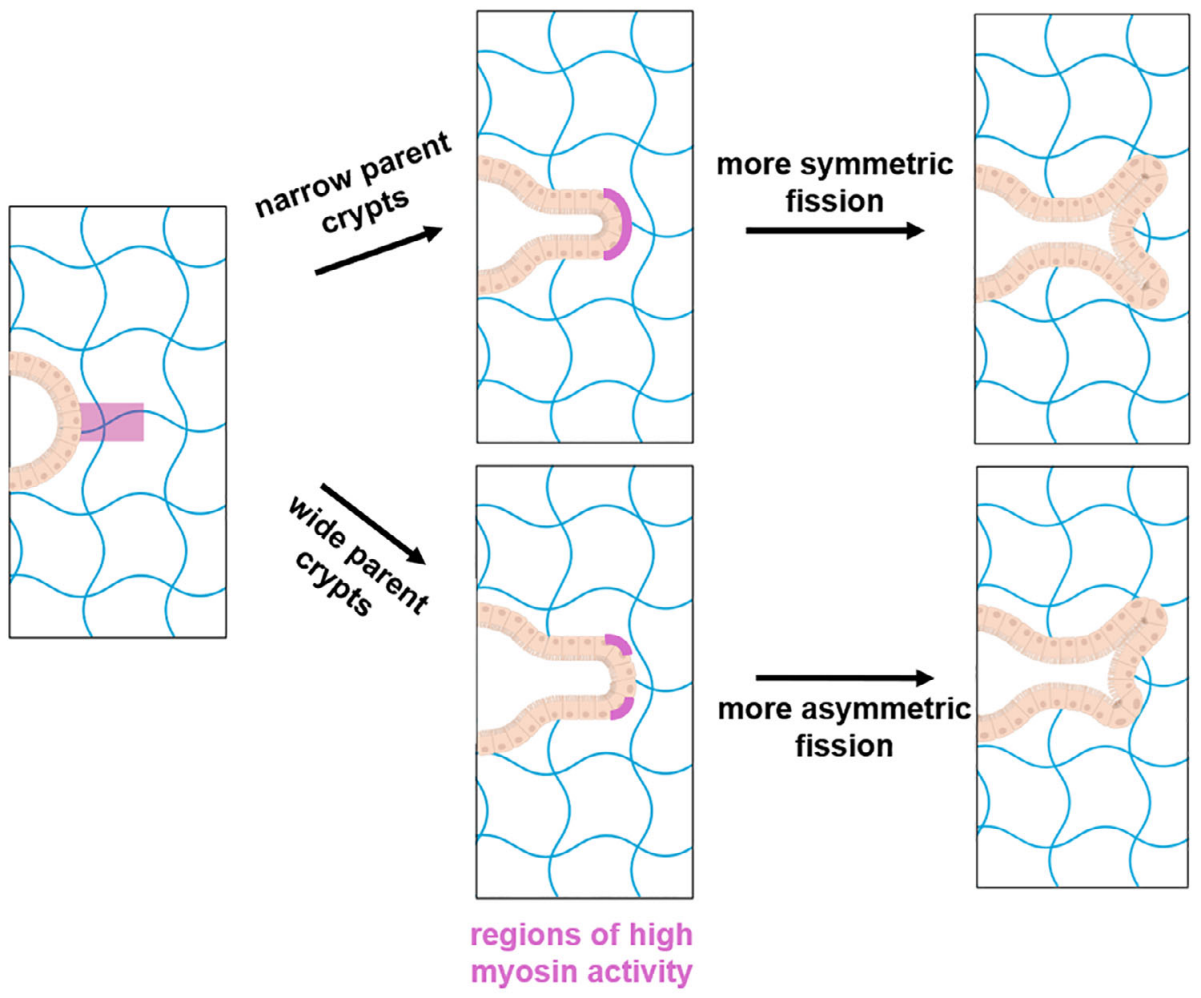
Spatiotemporally controlled crypt fission using photopatternable hydrogels reveals dependence of daughter symmetry on parent crypt shape.

Next, we asked what mechanosensitive machinery might link epithelial shape to symmetry outcomes using our controlled hydrogel system. We found that high curvature regions of the epithelium were enriched for active myosin (Figure 6), as detected by immunofluorescence staining for pMLC. Using tamoxifen‐inducible myosin perturbations, we implicate Myh9 as a broad regulator of daughter crypt symmetry, supporting a role for myosin‐dependent mechanosensing in controlling epithelial‐level homeostasis during crypt bifurcation. While myosin has previously been implicated in regulating crypt size [26] and extrusion of cells from the intestinal lining [32], our results extend its role to tissue‐level regeneration by revealing that actomyosin contractility can regulate how parent crypt shape controls daughter crypt symmetry.

Beyond pathological contexts, our findings can also have implications during healthy aging. It has long been recognized that crypt fission decreases with age [4, 37]. More recently, it has been reported that crypt size increases during aging, which has been linked to reduced myosin levels [26]. As such, our observation that higher parent crypt curvature is associated with higher fission symmetry suggests a potential route to improve crypt fission outcomes by controlling crypt dimensions and enhancing mechanosensing.

Future work could reveal how mechanical determinants identified here integrate with regenerative pathways like Wnt signaling [28, 38] to provide a more comprehensive understanding of physiological crypt fission regulation. Another signaling pathway of potential interest is the eph – ephrin communication between adjacent cells during tissue maintenance. It is known that the EphB‐expressing Paneth cells are correctly localized within the crypt region by repulsive interactions from ephrin‐B‐expressing villus resident cells [39]. Along these lines, disruption of eph–ephrin signaling altered Paneth cell positioning and subsequent fission symmetry within organoid crypts [19]. While we did not observe any difference in Paneth cell density between the two different daughter crypts after asymmetric fission, future studies could focus on how epithelial curvature‐dependent positioning of Paneth cells affects daughter crypt formation and fission symmetry. Also, it will be interesting to determine how these signaling inputs converge on mechanotransducers, such as the yes‐associated protein or YAP, during crypt bifurcation.

Our photoresponsive materials‐based approach to crypt fission can also be utilized for probing patient‐derived organoids [40] and is amenable to automated tissue engineering strategies [41]. Overall, our platform enables independent tuning of mechanical and biochemical cues during deterministic crypt fission in organoids, providing a route to identify mechanisms that support healthy homeostasis and potentially accelerate tissue regeneration.

## 4 Experimental Section

### 4.1 PEG Macromer Synthesis

Eight‐arm, 40‐kDa PEG‐8‐dibenzylcyclooctyne (DBCO) macromers were synthesized by coupling an 8‐arm PEG‐amine (0.5 g, 0.025 mmol, molecular weight (MW) = 40 000, JenKem USA) to a DBCO C6 acid (100 mg, 0.3 mmol, Click Chemistry Tools) using Hexafluorophosphate Azabenzotriazole Tetramethyl Uronium (HATU) (121 mg, 0.32 mmol, ChemPep) as an activator and N,N‐diisopropylethylamine as a base (0.175 mL, 1 mmol, Sigma–Aldrich). The reaction was purged with argon and left overnight to reach completion. The product was precipitated out of solution using three cold washes of diethyl ether to yield an off‐white solid. This solid was collected via centrifugation, dissolved in deionized (DI) water, and left to dialyze as previously described in Yavitt et al. [15].

Nitrobenzyl azide (NBA) ether‐functionalized PEG macromers was synthesized from 8‐arm 40 kDa PEG‐amine (1 g, 0.025 mmol, MW = 40 000, JenKem USA) via a coupling reaction with a fluorenylmethyoxycarbonyl (Fmoc)‐photolabile linker (218 mg, 0.42 mmol, Advanced ChemTech) with HATU (152 mg, 0.4 mmol, ChemPep) as an activator and 4‐methyl morpholine (107 mg, 0.8 mmol, Sigma–Aldrich) as a base. The reaction was left to purge under inert argon and reach completion overnight. Then, using cold washes of diethyl ether, the product was precipitated and collected using centrifugation. After collection, the product was dissolved in 80% N,N‐dimethylformamide (DMF), 20% piperidine to remove the Fmoc protecting group. The deprotection reaction was left for 24 h and then washed and collected with diethyl ether as described above. The product was once again dissolved in DMF to be coupled to azido butanoic acid (62 mg, 0.48 mmol), as outlined in DeForest et al. [42]. The final product was collected in cold diethyl ether, centrifuged, and subsequently dissolved in DI water. The product was dialyzed for 48 h (8 kDa MW cut‐off), then lyophilized to yield the final product.

### 4.2 Intestinal Stem Cell Culture and Organoid Maintenance

Animal procedures were approved by the Institutional Animal Care and Use Committee at the University of Colorado Anschutz Medical Campus (IACUC protocol no. 00084). All cells were cultured in humidified incubators at 37°C and 5% CO_2_. Murine small intestinal crypts were extracted from Defa4^Cre^; Rosa26^tdTomato^ mice as previously described [15]. For selected experiments, organoids were generated from crypts isolated from iMyh9 (Villin‐CreER;Myh9^flox/flox^;Rosa26^mTmG^) mice [26, 33, 43]. Organoids were encapsulated and grown in reduced growth factor Matrigel (Corning). Organoids were maintained in Advanced DMEM‐F12 (Invitrogen) containing, N‐2 and B‐27 supplements (Thermo Fisher Scientific), GlutaMAX (Gibco), 4‐(2‐hydroxyethyl)‐1‐piperazineethanesulfonic acid (HEPES, Gibco), and 1% penicillin–streptomycin (Gibco) supplemented with epidermal growth factor (EGF) (50 ng/mL, R&D Systems), noggin (100 ng/mL, PeproTech), R‐spondin conditioned medium (5% v/v, Organoid & Tissue Modeling Shared Resource, CU Anschutz), CHIR99021 (3 µM, Selleckchem), valproic acid (1 mM, Sigma–Aldrich), and N‐acetyl cysteine (1 mM, Sigma–Aldrich). “Base medium” refers to Advanced DMEM/F12 supplemented with N2 and B27 supplements, HEPES and penicillin–streptomycin, while “stem medium” refers to mase medium with EGF, noggin, R‐spondin, CHIR99021, valproic acid, and N‐acetyl cysteine (ENRCV+Nac). “Differentiation medium” refers to base medium with EGF, noggin, R‐spondin, and N‐acetyl cysteine (ENR+Nac). Media were changed every 2 days, and organoids were passaged by mechanical agitation every 6 days. In selected experiments, organoids were treated with 0.1 µM 4‐hydroxytamoxifen (4OHT, Stem Cell Technologies, 74052).

### 4.3 Organoid Isolation and Encapsulation Within Hydrogels

Organoids were dissociated into single cells using TrypLE Express Enzyme (Gibco) as outlined in Yavitt et al. [15]. From the single cells embedded in Matrigel domes, organoid colonies were grown for 3 days in stem medium. Once 3 culture days had passed, hydrogel solutions were made by using PEG‐8‐DBCO diluted in base medium. Into the precursor solution azide‐functionalized Arg‐Gly‐Asp (RGD, 0.8 mM) was added and allowed to react for 5 min. Next, laminin (0.14 mg/mL, Corning) was added to the solution on ice and mixed thoroughly. 3‐day‐old colonies were released from Matrigel using mechanical agitation from a pipette tip with cold base medium and centrifuged (100 g, 4 min) to form a pellet. Isolated organoid colonies were added to the chilled hydrogel solution, and PEG‐nitrobenzyl azide was added to the solution to initiate gelation. The final solution yielded a 2.5wt.% PEG composition that was vortexed for 3 s and pipetted onto thiol‐functionalized coverslips as 6 µL drops. These drops were then flattened using glass slides treated with Sigmacote (Sigma–Aldrich), separated by 250‐µm spacers placed over the coverslips. The gels were left sandwiched for 5 min at room temperature, after which the coverslips, attached to the gel, were separated from the glass slides. Gels were maintained in stem medium (300 µL) within tissue culture wells kept in incubators at 37°C and 5% CO_2_.

### 4.4 Photopatterning of Hydrogels

The day after encapsulation, individual coverslips with adhered gels were removed from tissue culture wells and were inverted onto a circular rubber stand for imaging and photopatterning. Photopatterning was done on gels one at a time utilizing a laser scanning confocal microscope (Zeiss LSM 710) fitted with 10×/0.5 numerical aperture (NA) air objective. For the primary patterning, one to two regions of interest (ROIs) of the appropriate width and length were scanned in a single plane around organoid colonies with a 405 nm laser operated at 40% laser power (1.79 mW), a pixel dwell of 12.64 µs, and a pixel size of 420 nm, resulting in a light dose of 12.8 J/cm^2^. This laser intensity allowed observable deformation of the epithelium while maintaining epithelial viability as demonstrated previously [13, 14, 15]. The laser power output was measured at the focal plane using a radiometer (Thorlabs). The angle between the two ROIs was chosen between 180 and 90 degrees based upon the empty space available around each organoid. About 10–20 organoid colonies were patterned per gel. The hydrogels were then returned to the tissue culture plate containing fresh differentiation medium. For the secondary crypt patterning, two ROIs of 40 µm width were arranged at the distal end of the first crypt in an orthogonal manner (90° angle between the two ROIs). These ROIs were scanned in an identical manner to the primary patterns, and the gel was returned to fresh differentiation medium. The patterned organoids were cultured for an additional 3 days before analysis.

### 4.5 Immunostaining

Samples were fixed with 4% paraformaldehyde for 20 min at room temperature (RT) with gentle rocking, followed by three consecutive 5‐min phosphate‐buffered saline (PBS) washes at RT. Samples were then treated with 10 mM sodium borohydride in PBS for 5 min at RT and washed twice for 5 min with PBS. Next, samples were permeabilized with 0.2% Triton‐X in PBS for 1 h at RT, followed by blocking with 5% goat serum and 0.01% Triton‐X in PBS blocking buffer for 1 h at room temperature. Post‐blocking, samples were treated overnight on a rocker at 4°C with primary antibody diluted in blocking buffer. The following day, samples were washed four times with PBS at 4°C, 1 h each time. Subsequently, samples were incubated overnight at 4°C with secondary antibody, 4',6‐diamidino‐2‐phenylindole (DAPI, 1:400), and in selected experiments with Alexa Fluor 488‐conjugated phalloidin (1:400, Invitrogen, A12379) diluted in blocking buffer.

Primary antibodies used were anti‐Olfm4 (D6Y5A) rabbit monoclonal antibody (1:200, Cell Signaling, 39141), anti‐Ki67 (SolA15) rat monoclonal antibody (1:200, Invitrogen, 14‐5698‐82), and anti‐phospho‐myosin light chain 2 (Ser19) rabbit antibody (1:100, Cell Signaling, 3671). Secondary antibodies used were Alexa Fluor 647 goat anti‐rabbit antibody (1:200; Invitrogen, A21245) and Alexa Fluor 488 goat anti‐rat antibody (1:200; Invitrogen, A11006).

### 4.6 Imaging

Phase images were acquired with 10×/0.25 NA and 20×/0.45 NA air objective lenses in an Olympus CKX53 microscope. Confocal images were obtained in a Nikon AXR laser scanning confocal microscope with 20×/0.95 NA water immersion objective. To visualize fluorescent signals, 405, 488, 561, and 647 laser lines were used.

### 4.7 Image Analysis

Image processing and analysis were conducted in ImageJ (National Institutes of Health) unless otherwise specified. Fidelity was calculated by measuring the length of the daughter crypt from the center of the bifurcation region to the end of the crypt. This value was then divided by the length of the secondary pattern that the organoid was exposed to and then multiplied by 100 to give a percentage.

Crypt efficiency was determined by manually counting the number of parent crypts that did bifurcate post‐secondary patterning and dividing it by the total number of parent crypts that underwent secondary patterning. The efficiency of each gel per given condition was then averaged for that experiment.

Daughter crypt analysis was conducted by measuring each crypt from the center of the bifurcation area to the end of the crypt. Lengths were corrected for images that were taken in higher magnification.

Symmetry analysis was done by finding the center of the bifurcation area and drawing a straight line from the center to the end of each individual crypt. The symmetry ratio was determined by taking the smaller crypt length over the longer crypt length, and ratios greater than or equal to 0.7 were classified as symmetric based upon Cheng et al. [28].

Parent crypt curvature was quantified using a modified MATLAB code reported in Yavitt et al. [15]. Briefly, DIC images of organoids prior to secondary patterning were used to manually trace the outer epithelial boundary. The traced boundary was then represented using a 10‐term (harmonics) Fourier series after normalization of boundary arc length. The local two‐dimensional curvature was calculated at each boundary point from the first and second derivatives of the fitted boundary, and parent crypt regions were identified as epithelial regions with positive curvature. For each parent crypt, a single representative curvature metric was defined as the arithmetic mean of the top 5% of positive curvature values within that crypt region, thereby capturing the most highly curved portion of the parent crypt. This value was then paired with the daughter‐crypt symmetry ratio measured for the corresponding fission event.

The arc length boundary normalization was achieved by calculating the perimeter of the organoid and normalizing to the maximum arc length θ, to 2π. Then, Fourier coefficients were determined using the following equations:

(1) $$x\left(\theta \right)=\frac{a_{0}}{2}+\sum_{n=1}^{10}\left(a_{n}\cos n\theta +b_{n}\sin n\theta \right)$$

(2) $$y\left(\theta \right)=\frac{c_{0}}{2}+\sum_{n=1}^{10}\left(c_{n}\cos n\theta +d_{n}\sin n\theta \right)$$

where *x* and *y* represent a Cartesian point on the boundary of the organoid, θ is the arclength at point (*x*, *y*), and *a*_0_, *a_n_*, *b_n_*, *c*_0_, *c_n_*, and *d_n_* are Fourier coefficients. The calculated Fourier coefficients provided a smooth parameterization or representation of the organoid boundary. Next, the curvature of the 2D organoid boundary was calculated using the following equation:

(3)

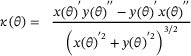

where *x*′ and *x*′′ are the first and second derivatives, respectively, of Equation (1) and *y*′ and *y*′′ represent the first and second derivatives, respectively, of Equation (2).

For pMLC intensity analysis, a midplane confocal image was selected for each organoid, and the epithelial boundary was manually traced using ImageJ. A custom Python script was used to fit a Fourier series to the traced boundary, compute pointwise curvature along the Fourier‐approximated contour, and quantify pMLC intensity along the boundary. For each point on the Fourier contour, the nearest pixel on the manually traced boundary was identified, and pMLC intensity was measured as the mean intensity within a 5 × 5 pixel window centered on that traced‐boundary pixel. Intensities were normalized to the mean boundary intensity for each organoid. Curvature values from all organoids in a specified condition were pooled, binned into equally spaced curvature intervals, and the mean normalized pMLC intensity within each curvature bin was plotted as a function of curvature.

To estimate the three‐dimensional epithelial surface area from two‐dimensional z‐stacks, the crypt epithelial boundary was detected in each z‐slice using the DAPI and lysozyme channels. Pixel intensities above the 97th percentile in the DAPI channel were clipped prior to normalization, after which the channel was normalized to the range [0, 1]. The lysozyme channel was independently normalized, enhanced using contrast‐limited adaptive histogram equalization (CLAHE), and thresholded to retain only pixels above the 95^th^ intensity percentile. The processed channels were combined and thresholded using Otsu's method, with the threshold scaled iteratively until the segmented region reached the image boundary to ensure complete segmentation of either parent or daughter crypts. The resulting segmentation was refined by removing small objects, applying morphological closing, filling holes, and retaining only the largest connected component. The largest contour by enclosed area was identified as the crypt epithelial boundary. Boundary shoulder points were identified, and a smoothing spline sampled at 5000 points was fit to the contour. This epithelial boundary length was measured for each z‐slice, and these measurements were numerically integrated over the z‐axis to estimate the epithelial surface area (µm^2^) of the crypt base:

(4) $$\mathrm{Surface}\;\mathrm{Area}\approx \int L\left(z\right)\mathrm{dz}$$

where *L*(*z*) is the epithelial boundary length at depth *z*. For each image, cells of the target cell type were manually counted from the TIFF stack. Cell number density was calculated by dividing the cell count by the calculated epithelial surface area.

### 4.8 Statistical Analysis

GraphPad Prism 8, 9, and 10 (GraphPad Software) was used for all statistical analyses. The Shapiro‐Wilk test was used to test normality when the number of data points was between 3 and 8. For experiments with >8 data points, the D'Agostino‐Pearson omnibus normality test was used to assess whether data were normally distributed. Analyses were performed using unpaired t tests, Mann‐Whitney tests, or Kruskal–Wallis tests followed by Dunn's test. For all data, *p* < 0.05 was considered significant.

## Author Contributions

D.L.M. designed the study, performed and analyzed the majority of the experiments, and wrote the manuscript. K.B. conceptualized, designed, and supervised the study, performed and analyzed selected experiments, synthesized macromers, and wrote the manuscript. N.R.P. and A.K. performed selected experiments, analyzed data, and edited the manuscript. D.C.‐A., O.D.K., and M.C. performed selected experiments. P.J.D. performed selected experiments, provided critical input, and edited the manuscript. K.S.A. conceptualized, designed, and supervised the study, provided critical input, and edited the manuscript.

## Conflicts of Interest

The authors declare no conflicts of interest.

## Supporting information




**Supporting File**: advs77397‐sup‐0001‐SuppMat.docx.

## Data Availability

The data that support the findings of this study are available from the corresponding author upon reasonable request.
