# Supplementary material for: Sequential Patterning of Photoresponsive Hydrogels Directs Crypt Fission and Reveals the Role of Epithelial Curvature on Fission Symmetry
Source: Adv Sci (Weinh). 2026 Aug 29;13(63):e77397. doi: 10.1002/advs.77397 (PMC13525618; doi:10.1002/advs.77397)
Supplement: Supplementary file 1 [file ADVS-13-e77397-s001.docx]

**Supplemental information** for

**Sequential Patterning of Photoresponsive Hydrogels Directs Crypt Fission and Reveals the Role of Epithelial Curvature on Fission Symmetry**

Delaney L. McNally ^1, #^, Kaustav Bera ^1, 2, #^, Nolan R. Petrich ^1, 2^, Alex Khang ^1, 2, 6^,
Marilyne Coulombe ^3^, David Castillo-Azofeifa ^4^, Ophir D. Klein ^4, 5^, Peter J. Dempsey ^3^,
Kristi S. Anseth ^1, 2^ *

^1^ Department of Chemical and Biological Engineering, University of Colorado Boulder, Boulder, CO, 80303 USA

^2^ BioFrontiers Institute, University of Colorado Boulder, Boulder, CO, 80303 USA

^3^ Section of Developmental Biology, Department of Pediatrics, University of Colorado, Denver, CO, 80204 USA

^4^ Program in Craniofacial Biology and Department of Orofacial Sciences, University of California, San Francisco, San Francisco, CA, 90089 USA

^5^ Department of Pediatrics, Cedars-Sinai Guerin Children's, Los Angeles, CA, 90048 USA

^6^ Present address: Allen Institute for Cell Science, Seattle, WA, 98109, USA

\# D.L.M. and K.B. contributed equally to this work and are co-first authors

*Correspondence: [kristi.anseth@colorado.edu](mailto:kristi.anseth@colorado.edu) (K.S.A)

**SI Figure 1:** Representative confocal images of DAPI and f-actin in both parent crypts (top) and daughter crypts (bottom) after patterning showing polarized epithelium after parent and daughter crypt formation. Scale = 50 μm.

**SI Figure 2:** **a**) Schematic representation of organoid patterning with one or two primary patterns to obtain one or two primary crypts, respectively. In some cases, the two rectangular regions (arms) were placed at 90 degrees instead of 180 degrees to each other. **b**) Quantification of viable crypts when one or two rectangular regions were patterned around an organoid to obtain one or two parent crypts, respectively. Statistical analysis was performed using unpaired t-test. **c**) Representative images of Defa4+ Paneth cell localization within organoids that were exposed to one or two primary patterns. Scale = 100 μm. **d**) Quantification of number of Paneth cells from panel b. Statistical analysis was performed using Mann-Whitney test.

**SI Figure 3:** Representation of daughter crypt efficiency and symmetry ratio analysis. Top panels show organoids that were classified as inefficient (left) and efficient (right). Bottom panels show symmetry ratio as percentage (SRP) measurements from the center of the bifurcation region. Blue lines represent length of each daughter crypt. The images show daughter crypts after (left) asymmetric fission with a SRP of 57.8% and (right) a symmetric fission event (right) with a SRP of 93.4%. Scale = 100 μm.

**SI Figure 4:** **a**) Quantification of Paneth Cell Density from both symmetric and asymmetric daughter crypts. **b, c**) Quantification of Paneth Cell Density from the longer and shorter crypts for symmetric and asymmetric crypts, respectively, from panel b. Statistical analyses were performed using unpaired t-tests. Data from ≥ 30 daughter crypt pairs, pooled from 2 independent experiments.

 **SI Figure 5:** **a)** Representative images of DAPI and Ki67+ cells in daughter crypts for both symmetric (top) and asymmetric (bottom) fission after patterning. Scale = 50 μm. **b**) Quantification of percentage of Ki67+ cells from both symmetric and asymmetric daughter crypts. **c, d**) Quantification of percentage of Ki67+ cells from the longer and shorter crypts for symmetric and asymmetric crypts, respectively, from panel b. Statistical analyses were performed using unpaired t-test. Data from ≥18 daughter crypt pairs, pooled from 2 independent experiments.

**SI Figure 6:** **a**, **b**) Normalized pMLC intensity along epithelial boundary plotted against local epithelial curvature of organoids exposed to 50 µm (a) or 70 µm (b) primary patterns. Data are the moving average ± s.e.m. for ≥20 organoids in each condition from 3 experiments.

**SI Figure 7:** Schematic outlines the experimental conditions and corresponding media types for experiments with iMyh9 organoids. Primary patterning was performed on day one and the secondary patterns were exposed on day four to initiate the crypt fission process. Organoids were grown till day seven for analysis. The medium was supplemented with 0.1 μM 4OHT starting on day three.
